# The impact of COVID-19 on individuals with ASD in the US: Parent perspectives on social and support concerns

**DOI:** 10.1371/journal.pone.0270845

**Published:** 2022-08-17

**Authors:** Emily Furar, Florence Wang, Jennifer S. Durocher, Yeojin A. Ahn, Idil Memis, Leylane Cavalcante, Lorena Klahr, Andrea C. Samson, Jo Van Herwegen, Daniel Dukes, Michael Alessandri, Rahul Mittal, Adrien A. Eshraghi

**Affiliations:** 1 Hearing Research and Communication Disorders Laboratory, Department of Otolaryngology, Miller School of Medicine, University of Miami, Miami, Florida, United States of America; 2 Department of Psychology, University of Miami, Coral Gables, Florida, United States of America; 3 Institute of Special Education, University of Fribourg, Fribourg, Switzerland; 4 Faculty of Psychology, Unidistance Suisse, Brig, Switzerland; 5 Department of Psychology and Human Development, UCL, Institute of Education, University College London, London, United Kingdom; 6 Swiss Center for Affective Sciences, University of Geneva, Geneva, Switzerland; 7 Department of Neurological Surgery, Miller School of Medicine, University of Miami, Miami, Florida, United States of America; 8 Department of Pediatrics, Miller School of Medicine, University of Miami, Miami, Florida, United States of America; University of Perugia: Universita degli Studi di Perugia, ITALY

## Abstract

The COVID-19 pandemic’s disruptions to daily routines and services have proven especially challenging for children with autism spectrum disorder (ASD) and their families. The current retrospective study aimed to determine the impact of the COVID-19 pandemic’s social environmental changes on parental ratings of personal and child concerns about family conflict, opportunities for social interaction, and loss of institutional support (school and therapy services). Analyses of responses from families with ASD in the US determined differences in concerns across three time points which were measured simultaneously: prior to COVID-19, at the start of COVID-19, and at the time of survey completion. From our sample of 246 school-aged children, parents retrospectively reported significantly increasing levels of concern for both themselves and their children over time, with parents’ personal concern levels rated consistently higher than their ratings of their child’s level of concern. Concerns about loss of institutional support were higher for parents of children reported as having co-occurring intellectual disability. Further, parents of younger children also reported more concerns about loss of services, as well as more social concerns. For parent ratings of child concerns, children who were reportedly aware of COVID-19 were determined to have higher levels of social concerns and concerns about loss of institutional support. Meanwhile, the child’s age and gender did not impact their parent ratings of child concerns. The increased level of parental and child-perceived concerns over the course of the pandemic suggests a need for improved service delivery and support for these families. The high levels of concerns observed in the current study provide support for the need to assess families’ priorities and tailor services to best meet families’ needs. This will potentially increase the quality of life of family members, and improve ASD services across the lifespan, and improve outcomes.

## Introduction

The COVID-19 pandemic has had far-reaching global impacts, resulting in dramatic changes to all aspects of daily life. COVID-19 was declared by the World Health Organization (WHO) as a global pandemic on March 11, 2020, and by the end of April, there were over 2.8 million infections and 200,000 deaths worldwide [1,2]. In the US alone, there were over 1 million confirmed cases by the end of April [1,2].

A large and growing body of literature has emerged on the challenges faced by families of children with developmental disabilities, including those with autism spectrum disorder (ASD), during the pandemic. Due to their social interaction and communication challenges, restricted and repetitive patterns of behaviors, interests, and activities, and co-occurring psychological and behavioral symptoms, children with ASD and their families are at significant risk for adverse effects from the pandemic [3–5]. Research has indicated that families of children with developmental disabilities and/or ASD have experienced higher levels of caregiver burden, parenting stress and psychological distress, increased anxiety, and an exacerbation of behavioral and psychiatric problems in children [6–16]. In addition, family disputes and conflict have also been found to worsen during the pandemic [17–22], however, this has been understudied in families of children with developmental disabilities [10,11].

COVID-19 related lockdown measures also resulted in widespread school closures across the globe [23,24]. For children with disabilities, this was experienced alongside a loss of access to special education services and private therapies [4,12–15,25,26]. Further, while most schools provided some level of remote or virtual learning opportunities [7], caregivers of individuals with ASD have generally rated the perceived benefit as low [12,13]. Concerns about regression during the pandemic were raised by many parents of children with ASD [27–29] and appear to be related more to the loss of social relationships and social skills than to concerns about attainment of academic skills [26].

Despite the growing body of literature on the impact of the COVID-19 pandemic on families of children with disabilities, only a limited number of studies have attempted to determine the effect of the pandemic specifically for families of children with ASD using a US cohort. As studies have shown that perceptions, and experiences, as well as the needs of parents of children with autism may vary across countries [30,31], there is a need to understand the effect of COVID-19 pandemic on social opportunities and loss of institutional support in US population. The availability of this information will help in better serving the needs of individuals with ASD and their families during future public health emergencies.

The current study has the potential to make several unique contributions to the literature. First, it will allow for the ability to evaluate how concerns change over the initial months of the pandemic for parents and children. Only one other study to date, using responses from the UK from the same larger international retrospective survey as the present study, has examined changes in parent concerns over three time points [10]. Additionally, while parent concerns regarding school closures and loss of therapeutic services have been well documented in families of children with disabilities [7,10–12,25,28,29], relatively few studies have examined child related concerns in this area [10,11,32]. Assessing parents’ perceptions of the concerns of their child with ASD will allow for better understanding of how the pandemic may affect individuals with ASD differently from their caregivers. Furthermore, only a handful of studies have specifically examined the social concerns of families of children with ASD during the COVID-19 pandemic, and none to our knowledge have examined family conflict in this group. This is an important undertaking given that social skills and interactions are a core area of difficulty and a main focus of intervention for this population, and family disputes and conflict may lead to increases in stress, psychological distress, and problematic child behaviors [17].

Finally, although there is an overwhelming evidence of the negative impact of the pandemic on families of children with disabilities, including those with ASD, there is limited information about child characteristics (such as age, gender, autism severity, intellectual disability, and COVID awareness) that might impact pandemic related concerns and outcomes. With respect to age, parents of younger children with ASD have been found to have higher levels of stress [12–14] and concerns about service disruptions [13]; younger children were also more likely to exhibit a worsening of behavior problems in one [8] but not in another [12] study. Parents of children with cognitive delays also had greater concerns about service disruptions [13] while greater ASD severity in children was associated with higher levels of parent stress [13,14]. As many children with ASD have deficits in abstract thinking, which make it difficult for them to understand the reasons for COVID-19 related social distancing and stay home orders [6,33], recent studies have included COVID-19 awareness questions within their surveys [10,11]. Emerging literature suggests that parents are more concerned about service disruptions for children with lower levels of COVID-19 understanding or awareness. Associations between awareness and behavioral and mental health outcomes are less clear. Greater COVID-19 awareness has been linked to higher levels of child anxiety and an increased risk for psychiatric problems in some studies [11,16], while other studies have shown no relationship with anxiety [10] or a decreased likelihood of emotional or mental health impacts from the pandemic [13]. Associations between COVID-19 awareness and parent-ratings of child concerns, however, have not yet been explored. Better understanding of the child characteristics that might predict social and family concerns of parents and children with ASD has implications for improving outcomes by allowing for modifications to the service system and targeted interventions to decrease parenting stress and improve the well-being of the whole family in the future.

Based on previous studies, we hypothesized that both parental concern ratings and the parent ratings of child concern would increase over time as the COVID-19 pandemic progressed. In addition, we also explored the effect of age on parent and perceived child concerns over time to determine whether pandemic-related service disruptions and social distancing regulations have had a differential effect on children with ASD based on age, as this area has not been adequately studied to date. It is our hope that any age-related differences can be addressed in future pandemics as well, in order to improve service delivery aimed at the varying needs and concerns of these children. Finally, we explored whether gender, co-occurring intellectual disability, and differences in COVID-19 awareness were related to parent and parent perceived child concerns.

Therefore, the objective of the current retrospective study is to determine how the COVID-19 pandemic’s resulting disruptions to the social environment of individuals with ASD have impacted both parental and perceived child concerns regarding family conflict, access to social interactions, and a loss of institutional (school and therapy) services for families in the US, based on responses from a survey. The current study aims to examine how parent and perceived child concerns change over three distinct time points, by assessing current (several months into the pandemic) as well as retrospectively rated (before the pandemic, and at the start of the pandemic) levels of concern. In addition, we also sought to understand whether child characteristics (such as age, gender, intellectual disability, and COVID-19 awareness) were associated with parental and parent-rated child concerns.

## Methods

### Survey

In the beginning of the COVID-19 pandemic, a survey entitled “COVID-19 Crisis Response Survey for Families of Individuals with Special Needs” was developed with the goal to better understand the impact of the pandemic on families with a child with special education needs and disabilities (SEND), including neurodevelopmental disorders. The questions about concerns were tapped into different domains such as interpersonal relationships, physical well-being, material well-being, and emotional well-being as described in previous studies [10,11]. Parents and caregivers were asked to report their own and their perception of their child’s anxiety and concerns at the time they were completing the survey (data in the US were collected between April 9^th^, 2020, and July 1^st^, 2020 in the US). At the same time, they were asked to retrospectively report their concerns for two other time points of interest: before the COVID-19 pandemic (prior to March 2020), at the start of the COVID-19 crisis (beginning in March 2020). The survey also contained open-ended questions to provide more information on their child’s situation or he or she dealt with stress.

This completely anonymous survey resulted from a multinational collaborative effort and was made available in 16 languages through an online platform (https://www.specialneedscovid.org/) and is fully available here: http://osf.io/5nkq9 DOI:10.17605/OSF.IO/5NKQ9. All collaborators disseminated the link within their own geographical area. Participation in the anonymous online study was entirely voluntary and not renumerated. Participants were informed about the goal of the online survey at the beginning of the study and were asked to proceed to begin the questionnaire in agreement that their data could be used for research purposes (informed consent was provided by clicking on the agreement button). At the very end of the survey, they were asked again if their provided responses could be used for research. In case they were not agreeing, their data was not included in the data analysis. The full survey can be found here: http://osf.io/5nkq9. The survey took about 30 minutes to complete and has been approved by the ethics committee of the Swiss Distance University Institute as of April 3, 2020 (Nr. 2020-03-00002).

For the purpose of this study, we selected the following items due to their relevance in the context of the social domain of ASD. Each of the following survey questions was rated on a scale from 1 to 5 for the current and the two retrospective time points, where 1 was not at all concerned and 5 was extremely concerned:

- “How concerned were/are you about the fact that your child has fewer occasions for social contact and interaction?”
- “How concerned was/is your child about not being able to meet peers and friends?”
- “How concerned were/are you that your child is not able to approach others?”
- “How concerned was/is your child about not being able to approach others?”
- “How concerned were/are you about family conflict?”
- “How concerned was/is your child about family conflict?”
- “How concerned were/are you about the loss of institutional support for your child including interventions?”
- “How concerned was/is your child about the loss of institutional support including interventions?”

### Participants

Participants of the survey included 309 caregivers/parents of individuals with ASD in the US, 273 of whom were women (88.9%), aged 22 to 73 years old (*M* = 47.1 years old; *SD* = 18.3 years). Of these participants, 244 (79.2%) had a university degree or equivalent. The majority of individuals with ASD included in this study were male (81.2%) and the overall mean age of the individuals with ASD was 13.9 years (*SD* = 8.1 years, range = 2–55 years). For this study, we selected only children in school-age from 3 to 21 years old (n = 246; age: *M* = 11.5 years old, *SD* = 4.9). Demographic data and clinical data were collected (Table 1). More than half of our sample (n = 131; 53.3%) were reported to have mild to severe intellectual disabilities. In addition to ASD, most participants also had an additional health (n = 115; 46.8%) or psychological condition (n = 92; 37.4%) such as anxiety disorder (18.7%), attention deficit hyperactivity disorder (17.1%), sleep disturbances (13.0%), depression (12.6%), obsessive compulsive disorder (2.9%), and bipolar disorder (0.4%). Additionally, 13 of them (5.3%) reported other psychological issues including personality disorder, affective disorder, communication disorder, dyspraxia, dyscalculia, or dyslexia. Before COVID-19, 98.4% (n = 242) of the children lived with their parents (versus 1.6% who lived in institutional or similar settings or on their own) and this proportion remained stable during COVID-19.

**Table 1:** Participant demographic characteristics and clinical information (*N* = 246).

	*N*	*%*
**Sex**		
Male	207	84.2
Female	39	15.9
**Age (years; mean, SD)**	**11.5**	**4.9**
3–9	100	40.7
10–15	92	37.4
Over 15	54	22.0
**Intellectual disabilities**		
None	103	41.9
Mild to moderate	102	41.5
Severe	29	11.8
ND	12	4.9
**ASD on medication**		
Yes	102	41.6
No	132	53.7
ND	12	4.7
**Other health issues** *	**115**	**46.8**
Epilepsy	68	28.6
Asthma	9	4.2
Cardiovascular conditions	33	13.4
Diabetes	3	1.2
Overweight	70	28.5
Other	44	17.9
**Other psychological disorders** *	**92**	**37.4**
Sleep conditions	32	13.0
ADHD	42	25.9
Anxiety disorder	46	18.7
Obsessive compulsive disorder	7	2.9
Bipolar disorder	1	0.4
Depression	31	12.6
Other	13	5.3

ADHD: Attention deficit hyperactivity disorder; ND: No data.

* They are not mutually exclusive.

Participants also reported whether their child was aware (n = 132; 53.7%) or not aware of COVID-19 (n = 46; 18.7%); this question was meant to assess whether the child understood that the changes to their routine and social distancing measures were due to the COVID-19 pandemic.

### Data analyses

For each analysis, we excluded participants with missing data using pairwise exclusions. A series of repeated measures analyses of covariance (ANCOVA) were performed in IBM SPSS Statistics Windows version 26.0.0. For parent concern ratings, we assessed overall differences in the mean ratings of social concerns across the three time points (Time 1 versus Time 2 versus Time 3) while controlling for child age at the time of the survey completion. The interaction between time point and child age for each parent social concern was also examined.

For parent-rated child social concerns, we conducted 3 (time points) x 2 (COVID-19 awareness: “aware” versus “not aware”) repeated measures ANCOVAs, with child age at the time of the survey completion as a covariate. The main effects of time point and COVID-19 awareness were examined. Additionally, we examined interaction effects between time point and child age, and between time point and COVID-19 awareness. Multiple comparisons were adjusted using Bonferroni corrections.

Finally, stepwise multiple linear regressions were performed to predict parent social concern ratings at the start of COVID-19 (time point 2) from child age and gender, and to predict parent-rated child social concerns at time point 2 from child age, gender, and COVID-19 awareness. Consistent with Sideropoulos et al. [10], we selected time point 2 (the beginning of COVID-19) to assess the level of concerns at the initiation of uncertain stressful event. Significant models and unique predictors were identified. We treated all concern ratings in all analyses as continuous variables to be consistent with previous studies [10].

## Results

### Preliminary analyses

Preliminary analysis of concern rating at each timepoint was conducted to assess the main effects of child intellectual disability (ID; ID vs. no ID), COVID-19 awareness (aware vs. not aware), and sex (male vs. female), as well as interaction effects. A child ID x COVID-19 awareness x sex analysis of concern rating indicated significant effects of ID on parent concern ratings about the loss of institutional support for the child at time point 3, *F*(1, 167) = 4.60, *p* = .03, η^2^p = .03. These findings suggest that parent ratings of their concerns about the loss of institutional support for the child at time point 3 was greater for parents of children with ID (*M* = 4.28, *SD* = 1.09, *n =* 98) than those of children without ID (*M* = 3.88, *SD* = 1.32, *n* = 76).

Moreover, there were significant effects of child’s COVID awareness on parent ratings of child concerns about lack of peer interaction at time point 2, *F*(1, 167) = 12.62, *p* < .001, η^2^p = .07, and at time point 3, *F*(1, 167) = 11.53, *p* = .001, η^2^p = .06. At time points 2 and 3, parents rated their child’s concern about the lack of peer interaction higher for children who were aware of COVID-19 (time point 2: *M* = 2.66, *SD* = 1.46, *n* = 130; time point 3: *M* = 3.11, *SD* = 1.47, *n* = 130) than for children who were not aware of COVID-19 (time point 2: *M* = 1.70, *SD* = 1.11, *n* = 44; time point 3: *M* = 2.07, *SD* = 1.30, *n* = 44). Similarly, there were significant effects of child’s COVID awareness on parent ratings of child concerns about inability to approach others at time point 2, *F*(1, 167) = 9.52, *p* = .002, η^2^p = .05, and at time point 3, *F*(1, 167) = 6.99, *p* = .01, η^2^p = .04, which indicated that parents rated their child’s concern about inability to approach others higher for children who were aware of COVID-19 (time point 2: *M* = 2.15, *SD* = 1.27, *n* = 130; time point 3: *M* = 2.48, *SD* = 1.40, *n* = 130) than for children who were not aware of COVID-19 (time point 2: *M* = 1.52, *SD* = 1.09, *n* = 44; time point 3: *M* = 1.77, *SD* = 1.31, *n* = 44). Additionally, parent-rated child concerns about family conflict at time point 2 were greater for children who were aware of COVID-19 (*M* = 1.82, *SD* = 1.26, *n* = 130) than those who were not aware (*M* = 1.36, *SD* = .87, *n* = 44), *F*(1, 167) = 6.03, *p* = .02, η^2^p = .03. Parent-rated child concerns about the loss of institutional support at time point 3 were also greater for children who were aware of COVID (*M* = 2.68, *SD* = 1.48, *n* = 130) than those who were not aware (*M* = 2.18, *SD* = 1.45, *n* = 44), *F*(1, 167) = 4.07, *p =* .05, η^2^p = .02. No other main or interaction effects were significant. There were no main effect or interaction effect of sex on any of the concern ratings, thus subsequent analyses excluded child sex.

Bivariate correlations indicated that parents of younger children reported greater levels of concerns about child’s lack of social contact and interaction at time point 1 (*r* = -.16, *p* = .03, *n* = 193) and at time point 2 (*r* = -.16, *p* = .03, *n* = 192), child’s inability to approach others at all time points (time point 1: *r* = -.24, *p* = .001, *n* = 193; time point 2: *r =* -.19, *p* = .01, *n* = 192; time point 3: *r* = -.14, *p* = .05, *n* = 193), and the loss of institutional support for the child at time point 1 (*r* = -.19, *p* = .01, *n* = 192) and at time point 2 (*r* = -.22, *p* = .002, *n* = 193). Child’s age was significantly associated with ID, *t*(232) = 3.05, *p* = .003, *d* = .40, which indicated that children with ID (*M* = 12.15, *SD* = 4.90, *n* = 131) tended to be older than children without ID (*M* = 10.20, *SD* = 4.73). Child’s age was also associated with child’s COVID-19 awareness, *t*(180) = -3.04, *p* = .003, *d* = .49, which indicated that children who were aware of COVID-19 (*M* = 12.25, *SD* = 4.67, *n* = 133) tended to be older than those who were not aware (*M* = 9.80, *SD* = 5.25, *n* = 49). A chi-square test of independence showed that there was a significant relationship between child ID and COVID-19 awareness, which indicated that children who were not aware of COVID-19 were more likely to have ID than those who were aware of COVID-19, χ^2^ (1, *N* = 182) = 7.77, *p* = .01, ϕ = -.21.

### Changes in social concerns

#### Parent concerns

Table 2 includes descriptive statistics of parent concern ratings at each time point.

**Table 2:** Descriptive statistics of parent concern ratings and parent-rated child concern ratings at each time point.

Type of concern			Before COVID-19 (Time 1)		At the start of COVID-19 (Time 2)		During COVID-19 (Time 3)		*p* Time 1/ Time 2	*p* Time 2/ Time 3	*p* Time 1/ Time 3
		*n*	Mean	*SD*	Mean	*SD*	Mean	*SD*			
*Parent Concerns*											
Child’s lack of social contact and interaction		188	2.63	1.27	3.42	1.24	3.96	1.19	<.01	<.01	<.01
Child’s inability to approach others		188	2.39	1.37	3.07	1.40	3.54	1.47	<.01	.03	<.01
Family conflict		188	1.96	1.24	2.44	1.48	2.63	1.60	<.01	<.01	<.01
Loss of institutional support for the child		188	2.30	1.37	3.66	1.26	4.09	1.23	.20	<.01	.02
*Parent-Rated Child Concerns*	Covid-19 Awareness										
Lack of peer interaction	Total	178	1.69	1.09	2.40	1.43	2.84	1.49			
	Aware	132	1.77	1.10	2.64	1.46	3.09	1.47	<.01	<.01	<.01
	Not aware	46	1.48	1.03	1.72	1.09	2.11	1.30			
Inability to approach others	Total	178	1.47	.86	1.98	1.25	2.29	1.40			
	Aware	132	1.47	.80	2.14	1.27	2.46	1.40	<.01	.03	<.01
	Not aware	46	1.46	1.05	1.54	1.07	1.78	1.28			
Family conflict	Total	178	1.48	.95	1.69	1.18	1.82	1.29			
	Aware	132	1.57	1.01	1.80	1.25	1.94	1.34	.17	.44	.02
	Not aware	46	1.24	.71	1.35	.85	1.48	1.07			
Loss of institutional support	Total	178	1.44	.93	2.13	1.37	2.55	1.47			
	Aware	132	1.47	.98	2.24	1.40	2.67	1.47	.01	<.01	<.01
	Not aware	46	1.35	.79	1.80	1.22	2.20	1.42			

**Fig 1:**
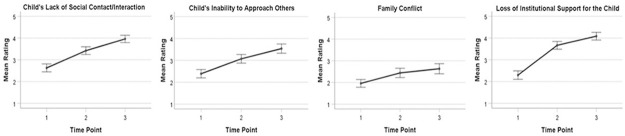
Line plots of estimated marginal means of the parent concern ratings. The plots demonstrate a significant linear change in all parent concern rating items over time. Time point 1: Before COVID-19; Time point 2: At the start of COVID-19; Time point 3: During COVID-19 (at the time of survey completion).

A series of repeated measures ANCOVA revealed that there was a significant main effect of time point for all parent social concern items (Table 3). That is, when the child age was controlled for, there was a significant linear increase over time in parent ratings of their concerns about the child’s lack of social contact, *F*(1, 186) = 16.97, *p* < .001, η^2^p = .08, the child’s inability to approach others, *F*(1, 186) = 9.36, *p* = .003, η^2^p = .05, family conflict, *F*(1, 186) = 11.07, *p* = .001, η^2^p = .06, and the loss of institutional support for the child, *F*(1, 186) = 22.04, *p* < .001, η^2^p = .11 (Fig 1). Mauchly’s Test of Sphericity indicated that the assumption of sphericity had been violated for all parent social concerns, thus the degrees of freedom were adjusted by using Huynh-Feldt correction for the ratings of parent concerns about the child’s lack of social contact, family conflict, and loss of institutional support for the child (Table 3). The degrees of freedom for ratings of parent concern about the child’s inability to approach others were adjusted using Greenhouse-Geisser correction. Post-hoc comparisons indicated significant differences in all parent social concern items between all three time points (S1 Table). There was no interaction effect of time point by child age on the parent concern ratings (Table 3).

**Table 3:** Sphericity violations and ANCOVA for ratings of social concerns.

Type of concern	Test of sphericity checks	Adjustment method and ε level	Source	df1	df2	*F*	*p*	η^2^p
*Parent Concerns*								
Child’s lack of social contact and interaction	*χ*^*2*^(2) = 36.18, *p*<.001	Huynh–Feldt correction (ε = .86)	Time	1.72	320.21	12.03	<.001	.06
			Time x Age	1.72	320.21	.32	.70	.002
Child’s inability to approach others	*χ*^*2*^(2) = 55.18, *p*<.001	Greenhouse-Geisser correction (ε = .79)	Time	1.59	295.73	7.03	.002	.04
			Time x Age	1.59	295.73	1.53	.22	.01
Family conflict	*χ*^*2*^(2) = 31.57, *p*<.001	Huynh–Feldt correction (ε = .88)	Time	1.75	326.04	7.24	.001	.04
			Time x Age	1.75	326.04	.37	.66	.002
Loss of institutional support for the child	*χ*^*2*^(2) = 27.93, *p*<.001	Huynh–Feldt correction (ε = .89)	Time	1.78	330.91	19.92	<.001	.10
			Time x Age	1.78	330.91	1.89	.16	.01
*Parent-Rated Child Concerns*								
Lack of peer interaction	*χ*^*2*^(2) = 21.58, *p*<.001	Huynh–Feldt correction (ε = .92)	Time	1.84	319.93	5.96	.004	.03
			COVID-19 awareness	1	174	.85	.36	.005
			Time x Age	1.84	319.93	.71	.48	.004
			Time x COVID-19 awareness	1.84	319.93	1.61	.20	.01
Inability to approach others	*χ*^*2*^(2) = 23.42, *p*<.001	Huynh–Feldt correction (ε = .91)	Time	1.82	317.23	2.05	.13	.01
			COVID-19 awareness	1	174	.29	.59	.002
			Time x Age	1.82	317.23	1.86	.16	.01
			Time x COVID-19 awareness	1.82	317.23	.97	.37	.01
Family conflict	*χ*^*2*^(2) = 19.98, *p*<.001	Huynh–Feldt correction (ε = .93)	Time	1.85	322.33	2.59	.08	.01
			COVID-19 awareness	1	174	.02	.89	1.04e-4
			Time x Age	1.85	322.33	.05	.94	3.11e-4
			Time x COVID-19 awareness	1.85	322.33	1.25	.29	.01
Loss of institutional support	*χ*^*2*^(2) = 24.07, *p*<.001	Huynh–Feldt correction (ε = .91)	Time	1.82	316.29	11.31	<.001	.06
			COVID-19 awareness	1	174	1.25	.27	.007
			Time x Age	1.82	316.29	.36	.68	.002
			Time x COVID-19 awareness	1.82	316.29	.89	.40	.01

χ2 = Chi-square test; df = degrees of freedom; ε = epsilon; η2p = partial eta squared.

### Parent-rated child concerns

**Fig 2:**
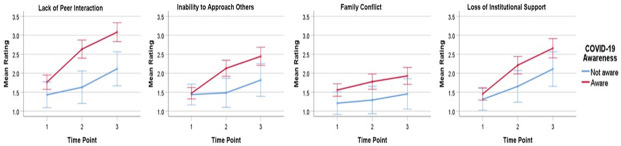
Line plots of estimated marginal means of the parent-rated child concern ratings. The plots show a significant linear change in the parent-rated child concerns about the lack of peer interaction and the loss of institutional support over time. The blue line represents children who were *not* aware of COVID-19, and the red line represents children who were aware of COVID-19. Error bars indicate 95% confidence intervals. Time point 1: Before COVID-19; Time point 2: At the start of COVID-19; Time point 3: During COVID-19 (at the time of survey completion).

There was a significant main effect of time point for the parent-rated child concerns regarding the lack of peer interaction, *F*(1.84, 319.93) = 5.96, *p* = .004, η^2^p = .03, and loss of institutional support, *F*(1.82, 316.29) = 11.31, *p* < .001, η^2^p = .06. However, there was no significant main effect of time point for the parent-rated child concerns about their inability to approach others and family conflict (Table 3). Moreover, there was no significant interaction between time point and child age, and between time point and child COVID-19 awareness, for all parent-rated child concern items. That is, when the child age was controlled for, there was a significant linear increase over time in parent-rated child concerns about the lack of peer interaction, *F*(1, 174) = 8.34, *p* = .004, η^2^p = .05, and the loss of institutional support, *F*(1, 174) = 16.56, *p* < .001, η^2^p = .09, for all children regardless of whether they were aware of COVID-19 (Fig 2). Mauchly’s Test of Sphericity indicated that the assumption of sphericity had been violated, thus the Huynh-Feldt corrections were used to adjust the degrees of freedom for all ratings of child concern items. Results of post-hoc comparisons corroborated that the parent-rated child concerns about the lack of peer interaction and loss of institutional support significantly increased from time point 1 to time point 2 and from time point 2 to time point 3 (S2 Table). For all parent-rated child concern items, there was no significant main effect of child COVID-19 awareness (Table 3).

### Multiple regressions to predict social concern levels at the beginning of COVID-19

#### Parent concerns

Initial regression models included child age as a potential predictor of parent ratings of social concerns at time point 2 (at the beginning of COVID-19). The potential predictors that had a significant univariate association (*p* < .05) with the parent concern rating were entered into the model with a stepwise approach. In the final models, child age was a unique predictor of parent ratings of concerns about the child’s lack of social contact, adjusted *R*^*2*^ = .02, *F*(1, 190) = 3.78, *p* = .03, inability to approach others, adjusted *R*^*2*^ = .03, *F*(1, 190) = 7.37, *p* = .01, and the loss of institutional support for the child, adjusted *R*^*2*^ = .04, *F*(1, 191) = 9.84, *p* = .002 (Table 4). Parent concern ratings of the child’s lack of social contact, inability to approach others, and the loss of institutional support for the child were greater for parents with younger children (*β* = -.04, *t*(190) = -2.25, *p* = .03; *β* = -.06, *t*(190) = -2.71, *p* = .01; *β* = -.06, *t*(191) = -3.14, *p* = .002, respectively). No unique predictor was attained for the parent concern rating of family conflict.

**Table 4:** Final regression models: Child age predicting parent concern ratings and child COVID-19 awareness predicting parent ratings of child concerns.

Type of concern	Model	B	Std. Error	β	*t*	*p*	*F*	R^2^	Adjusted R^2^	*p*
*Parent concerns*										
Child’s lack of social contact and interaction	(Constant)	3.91	.22	-	17.61	<.001	5.06	.03	.02	.03
	Child age	-.04	.02	-.16	-2.25	.03				
Child’s inability to approach others	(Constant)	3.70	.25	-	-	<.001	7.37	.04	.03	.01
	Child age	-.06	.02	-.19	-2.71	.01				
Loss of institutional support for the child	(Constant)	4.32	.22	-	19.57	<.001	9.84	.05	.04	.002
	Child age	-.06	.02	-.22	-3.14	.002				
*Parent-rated child concerns*										
Lack of peer interaction	(Constant)	.88	.41	-	2.12	.04	14.31	.07	.07	<.001
	COVID-19 awareness	.88	.23	.27	3.78	<.001				
Inability to approach others	(Constant)	1.07	.37	-	2.88	.004	6.45	.03	.03	.01
	COVID-19 awareness	.53	.21	.19	2.54	.01				

*Note*. Results of stepwise regression employing a *p* < .05 inclusion criterion.

#### Parent perception of child concerns

Initial regression models included child’s age and COVID-19 awareness as potential predictors of the parent ratings of child concerns. In the final models, the child COVID-19 awareness was a unique predictor of parent-rated child concerns about their lack of peer interaction, adjusted *R*^*2*^ = .07, *F*(1, 180) = 14.31, *p* < .001, and their inability to approach others, adjusted *R*^*2*^ = .03, *F*(1, 178) = 6.45, *p* = .01 (Table 4). Children who were aware of COVID-19 had greater levels of parent-reported child concerns about the lack of peer interaction and inability to approach others (*β* = .88, *t*(190) = 3.78, *p* < .001; *β* = .53, *t*(178) = 2.54, *p* = .01, respectively). No unique predictor was attained for the parent-rated child concerns about family conflict and the loss of institutional support.

## Discussion

The present retrospective study examined the impact of the first several months of the COVID-19 pandemic on the concerns and worries of families of individuals with ASD in the US. Four specific areas of concern were evaluated in caregivers: concern that their child had fewer opportunities for social contact and interactions (or not being able to meet peers and friends), concern that their child was not able to approach others, concern about family conflict, and concern about the loss of institutional support (school and therapeutic interventions), for their child. Parents also rated the degree to which they felt that their child was concerned about these same issues.

Changes in the level of concerns/worries were evaluated at 3 different time points of which the first two were retrospectively reported (before or pre-COVID-19, at the start or beginning of the pandemic, and several months into the pandemic which was the time at which the survey was completed), among children who were “aware” or “not aware” of the COVID-19 pandemic. Our investigation adds to previous studies in this area by expanding analyses to specifically look at trends in how both parent and parent perceived child concerns evolved over three time points during the pandemic in the US population. Further, this study is only one of a few investigations that have examined concerns regarding family conflict and loss of social opportunities, and the only to our knowledge to examine these concerns specifically in a sample of families of children with ASD using the US cohort. Finally, we add to an emerging literature on predictors of pandemic-related outcomes, by examining whether there are any significant differences based on child gender, age, co-occurring intellectual disability, or COVID-19 awareness status in parent and/or parent-rated child social concerns and concerns regarding family conflict, an area that has not yet been studied.

### Changes in parent concerns due to COVID-19

As hypothesized, parents reported clear and significantly increasing levels of concern over the beginning months of the pandemic. After controlling for age, there were significant increases in concerns at each time point regarding their child’s loss of institutional (school and therapeutic) support, loss of opportunities for social interactions, restrictions on being able to approach others, and family conflict.

Findings regarding concerns about loss of institutional support are consistent with previous literature which indicates significant concerns regarding regression in skills and an increase in behavioral challenges due to decreased access to educational services and behavioral interventions as a result of the pandemic [4,12,25,27,28,34]. After controlling for age, we found increasing levels of parent concern regarding lack of social opportunities for their children. This is an important finding, given that social deficits are a core symptom area within ASD; thus, it is not surprising that caregivers would be worried about the loss of social opportunities, regardless of their child’s age, as the pandemic progressed. Social distancing measures may lead to regression or difficulty developing and maintaining social and communication skills, since interpersonal interactions are important for practicing these skills, as well as social isolation, the loss of friendships, and increased boredom and loneliness [35]. Concerns regarding their child’s ability to navigate social situations after the pandemic when previously established social skills and norms may no longer apply [36] may also account for increased concerns regarding social approach in the present study.

A novel finding is that parents reported significantly increased levels of concern regarding family conflict over the course of the pandemic. While this finding differs from results of the same international study using a sample of families of children with disabilities from China [11], it is consistent with research documenting increases in family disputes, parent-child conflict, and child discipline among families in the general population [17–22] and those with children with disabilities in the UK [10] due to stay-at-home policies. Our results therefore extend these findings specifically to families of children with ASD. This is important as it has implications for future pandemics and public health emergencies. Social distancing guidelines create a high potential for conflict due to the significant amount of time members of the household spend together in close physical proximity while under increased general stress due to the pandemic [17]. Increased problem behaviors in their children with ASD due to the abrupt changes caused by the pandemic, and challenges in their children’s ability to independently access online learning, may also increase the likelihood of family conflict for this population [8,12,36].

### Changes in parent-perceived child concerns due to COVID-19

Also consistent with our hypotheses, parent ratings of perceived child concerns increased significantly at each of the pandemic-related time points. Specifically, parents indicated increased child concerns with respect to loss of peer interactions and loss of institutional support (e.g., school and therapeutic services), but did not report increased child concerns for family conflict or ability to approach others. The finding of parent perceived child concerns about a loss of social opportunities is an important addition to the literature, as it challenges preconceptions that individuals with ASD may not be motivated by social interactions [37–39]. This finding also contributes to an emerging literature that documents how the abrupt COVID-19 restrictions have had a considerable negative impact on social wellbeing for individuals with ASD with several studies noting that individuals with ASD missed social connections and friendships [10,32,35]. Likewise, parent perceived child concerns regarding a loss of institutional support may reflect a loss of social opportunities which are available within those settings. It may also be that parent ratings of children’s concerns are influenced by struggles with accessing virtual school and therapy services, since online schooling models are not optimal for individuals with ASD due the higher level of independence required compared to in-person schooling [12,40]. These considerations may be important to consider for children with ASD during any future disruptions to services due to a resurgence of the COVID-19 or another pandemic, as it will be critical to institute measures to maintain social connections and opportunities for children with ASD.

It should be noted that parents generally reported higher levels of pandemic-related concern in comparison to their ratings of their child’s concerns. Since parents, rather than the children themselves, were responding to the questions, it is not clear whether findings reflect true levels of child concerns. Parents could be under-reporting their child’s level of concern. Lower ratings of child concern may reflect parent’s perceptions of their child’s level of social motivation or ability to think or worry about these issues, rather than the child’s actual level of concern. However, findings may reflect true differences in the level of pandemic-related concerns for children compared to parents. It is also important to consider that reduced social demands during the pandemic may be viewed as beneficial by some children with ASD, resulting in lower levels of social concerns due to a reduction in experiences of social stigma, discrimination, and bullying, and a decreased need to engage in social camouflaging [41–43]. Therefore, strategies to address loss of social opportunities for children with ASD should take into account the child’s perspectives and concerns, so that strategies can be individualized to children’s needs. Further, the finding that child concerns about family conflict did not increase during the pandemic may reflect greater opportunities to bond and engage in leisure activities with their families, leading to decreased stress and increased closeness for some children [29,32,43].

### Predictors of parent and perceived child concerns

We initially examined whether concerns varied according to child age, gender, presence of co-occurring intellectual disability and COVID-awareness status. No differences in parent or parent-rated child concerns were observed for gender. Parent concerns regarding loss of institutional support were higher for children with intellectual disability, as well as for younger children. Parents of younger children also had higher concerns regarding loss of social opportunities for peer interaction and ability to approach others. Further, parents rated child concerns higher for children who were aware of COVID-19 with respect to loss of services, social opportunities and family conflict. These results add to our knowledge of how child characteristics may impact the concerns of families of children with ASD and will help practitioners better assess risk for negative outcomes and plan for prevention and intervention efforts in the future.

We also conducted multiple regression analyses to further explore which factors predicted concerns at the beginning of the pandemic (time 2). For parents, child age was a unique predictor of concerns. Specifically, concerns were greater for parent of younger children with respect to loss of social opportunities, inability to approach others, and loss of institutional support, but not for family conflict. These findings are consistent with research that suggests that parents of younger children may generally experience higher levels of stress relative to parents of older children [44–46]. Our findings also expand upon an emerging literature that shows that parents of younger children with ASD experience greater negative impacts of the pandemic and concerns about service disruptions [8,12–14], and extend these impacts to increased social concerns. Therefore, families of younger children with ASD may be more sensitive to rapid increases in stress and worry when faced with a loss of critical services, such as during the COVID-19 pandemic.

Previous research also suggests that parents of younger children with ASD have higher levels of stress regarding children’s social relatedness [46,47] which may have contributed to the current findings. In addition, social and verbal initiation are often noted as points of difficulty for children with ASD [48], and therapies and educational programs for younger children often include goals surrounding the development of social and communication skills [49]. Since these goals require the opportunity to approach and interact with others, social distancing and isolation guidelines may have a greater perceived impact for younger children whose parents are more likely to be concerned about possible regression or an inability to make progress in these developmental areas [26]. The literature also suggests that support needs and services for children with ASD appear to vary with child age, with younger children with ASD requiring more support and direct services than older children and receiving a wider range or variety of services which focus on speech, language and communication [49,50]. As early intervention is emphasized as critically important for younger children, any loss of services will likely create significant levels of concern. These findings are important because they specifically highlight that social concerns will need to be considered for children with ASD during future events resulting in service disruptions, which may be particularly salient and detrimental for younger children as well as those with co-occurring intellectual disability, so that interventions can be put in place maintain children’s social connections and ability to practice important social skills.

For children, COVID-19 awareness was a unique predictor of parent-rated child concerns. Children who were aware of COVID-19 had greater levels of parent-reported child social concerns (lack of peer interaction and inability to approach others). Children with less awareness of their social surroundings may have more difficulty articulating their concern, possibly influencing parents’ perceptions of their distress and leading to under-reporting in this group. It may also be that peer relationships (versus parent-child or sibling interactions) have a greater relative importance for individuals with higher overall levels of situational awareness, leading to a greater impact when they are not able to access established social relationships.

COVID-19 awareness did not, however, predict parent-rated concerns about family conflict or a loss of institutional support. Further, factors such as child age and gender did not seem to influence parent-rated child social concerns. These findings are consistent with those from Sideropoulos et al. [10] who found that COVID-19 awareness, but not gender or age, predicted anxiety levels for children with disabilities at the start of the pandemic.

The findings of our study suggest that careful clinical attention should be paid to the mental health of parents of children with developmental disorders [51]. Continuous efforts have evolved in order to provide clinical support to individuals with developmental needs, including those individuals with ASD. Physical distancing and service disruptions have led to increased worries for individuals with ASD and their caregivers, which create a need for increased support and the development of COVID-19 specific guidelines for intervention [3,4].

Researchers have recommended for such children and families to access quality telehealth therapy services [4] as an alternative to alleviate the loss of in-person support and services. However, some research also shows that the perceived benefit of telehealth is low especially among younger children [12]. Since past studies have correlated satisfaction regarding services with parental well-being [52], it may be particularly beneficial to target improvements in the delivery of support and services for this cohort during the pandemic. In this respect, interventions aimed at coaching parents may need to be developed and implemented for those who are not benefitting from virtual services. In addition, parents of younger children had the greatest increase in concerns about social interaction and contact at the start of the pandemic–a group that may not be able to access sources of social contact via virtual means. Careful attention should be paid to the social needs of this population during the pandemic through the development of creative ways to foster access to social and play partners.

### Limitations

This study offers important information on the impacts of COVID-19 on both parent and child social concerns within the US ASD community. It is important to note a clear limitation, however, which resides in the fact that child concern levels were rated by parents and not the individual with ASD themselves. While parent perceptions of concerns offer valuable insight for research, they cannot be considered a perfect proxy for understanding the lived experiences and mindsets of individuals with ASD. Given previous research as well as our current findings which suggest a correlation between parent concerns and ratings of child concern, parents could over or underestimate true child concerns [10,20]. Especially for those children with ASD who have limited spoken language and/or moderate to severe intellectual disability, it can be difficult for parents to recognize the extent of their child’s worries, either because the individual cannot provide input into their own mental or emotional process or because others have preconceived notions that they will be unable to. Too little research has evaluated concerns from the perspective of the person with ASD themselves, particularly for those who have more limited verbal and cognitive abilities [53]. Additionally, there are concerns of sample representativeness, given the fact that the majority of parent participants were highly educated, with a university degree or equivalent education level. Furthermore, we did not have data on family ethnicity/race or income level, which further limits representativeness of the sample. Due to the retrospective nature of the survey, there is also a possibility of recall bias in parent reporting. For example, studies have shown greater recall bias from participants with poorer outcomes, as well as decreased ability to recall accurately over longer time periods [54]. As this survey only required parents to recall information and emotions from only a few months prior, this is not likely to have as large of an effect on our study as it would have if the survey was completed further from the start of the pandemic. However, individuals who are more highly educated have performed better to a certain degree in recall, thus improving the confidence of our data’s reliability [55]. With these limitations, future research should include direct assessment of the experiences of individuals with ASD themselves, as well as attempting to include a more socioeconomically diverse population. It may also be beneficial to perform longitudinal studies in future studies as opposed to obtaining retroactive data in order to eliminate the possibility of recall bias.

### Conclusions

The information gathered from this study can be useful in determining effective techniques to support individuals with ASD and their families during such health emergencies due to the significant impact the pandemic has had on well-being and worries. Recent studies have recommended the implementation of mental health services tailored to individuals with ASD and their families [8,56]. By using this data, specialists can place a focus on these areas of increased stress delineated through this study, such as family conflict and access to social opportunities, in order to guide stress-mitigation techniques and inform modifications to autism therapies and services. The high levels of concerns highlighted in the current study provide support for the need to assess families’ priorities and tailor services to best meet families’ needs; this will potentially increase the quality of life of family members, and improve ASD services across the lifespan, and improve outcomes [57].

## Supporting information

S1 Table Post-hoc pairwise comparisons on the parent concern ratings. Pairwise comparisons based on estimated marginal means of parent concern ratings. Multiple comparisons were adjusted using the Bonferroni method. Time point 1: Before COVID-19; Time point 2: At the start of COVID-19; Time point 3: During COVID-19 (at the time of survey completion).
(DOCX)

S2 Table Post-hoc pairwise comparisons on the parent ratings of child concerns by child COVID-19 awareness. Pairwise comparisons based on estimated marginal means of parent ratings of child concerns. Multiple comparisons were adjusted using the Bonferroni method. Time point 1: Before COVID-19; Time point 2: At the start of COVID-19; Time point 3: During COVID-19 (at the time of survey completion).
(DOCX)
