# Supplementary material for: The impact of COVID-19 on individuals with ASD in the US: Parent perspectives on social and support concerns
Source: PLoS One. 2022 Aug 17;17(8):e0270845. doi: 10.1371/journal.pone.0270845 (PMC9384980; doi:10.1371/journal.pone.0270845)
Supplement: S1 Table — Pairwise comparisons based on estimated marginal means of parent concern ratings. Multiple comparisons were adjusted using the Bonferroni method. Time point 1: Before COVID-19; Time point 2: At the start of COVID-19; Time point 3: During COVID-19 (at the time of survey completion). (DOCX) [file pone.0270845.s001.docx]

| **Table S1**. Post-hoc pairwise comparisons – effect of time point on the parent concern ratings | | | | | | | |
| --- | --- | --- | --- | --- | --- | --- | --- |
| Type of concern | Time point (I) | Time point (J) | Mean Difference (I-J) | *SE* | *p* | 95% CI Lower Bound | 95% CI Upper Bound |
| Lack of social support and interaction | 1 | 2 | -.79 | .09 | <.001 | -1.01 | -.58 |
|  | 1 | 3 | -1.33 | .11 | <.001 | -1.60 | -1.06 |
|  | 2 | 3 | -.54 | .08 | <.001 | -.73 | -.35 |
| Child’s inability to approach others | 1 | 2 | -.68 | .08 | <.001 | -.87 | -.49 |
|  | 1 | 3 | -1.15 | .10 | <.001 | -1.40 | -.90 |
|  | 2 | 3 | -.47 | .07 | <.001 | -.63 | -.30 |
| Family conflict | 1 | 2 | -.48 | .06 | <.001 | -.63 | -.34 |
|  | 1 | 3 | -.68 | .09 | <.001 | -.89 | -.46 |
|  | 2 | 3 | -.19 | .08 | .049 | .00 | .38 |
| Loss of institutional support for the child | 1 | 2 | -1.37 | .09 | <.001 | -1.59 | -1.15 |
|  | 1 | 3 | -1.79 | .12 | <.001 | -2.07 | -1.50 |
|  | 2 | 3 | -.42 | .09 | <.001 | -.64 | -.20 |
| *Note.* Pairwise comparisons based on estimated marginal means of parent concern ratings. All mean difference is significant at the .05 level. Multiple comparisons were adjusted using the Bonferroni method. Time point 1: Before COVID-19; Time point 2: At the start of COVID-19; Time point 3: During COVID-19 (at the time of survey completion). | | | | | | | |
