# Supplementary material for: The impact of COVID-19 on individuals with ASD in the US: Parent perspectives on social and support concerns
Source: PLoS One. 2022 Aug 17;17(8):e0270845. doi: 10.1371/journal.pone.0270845 (PMC9384980; doi:10.1371/journal.pone.0270845)
Supplement: S2 Table — Pairwise comparisons based on estimated marginal means of parent ratings of child concerns. Multiple comparisons were adjusted using the Bonferroni method. Time point 1: Before COVID-19; Time point 2: At the start of COVID-19; Time point 3: During COVID-19 (at the time of survey completion). (DOCX) [file pone.0270845.s002.docx]

| **Table S2**. Post-hoc pairwise comparisons – effect of time point on the parent ratings of child concerns by child COVID-19 awareness | | | | | | | | |
| --- | --- | --- | --- | --- | --- | --- | --- | --- |
| Type of concern | COVID-19 awareness | Time point (I) | Time point (J) | Mean Difference (I-J) | *SE* | *p* | 95% CI Lower Bound | 95% CI Upper Bound |
| Lack of peer interaction | Aware | 1 | 2 | -.87* | .10 | <.001 | -1.12 | -.62 |
|  |  | 1 | 3 | -1.32* | .12 | <.001 | -1.61 | -1.02 |
|  |  | 2 | 3 | -.45* | .09 | <.001 | -.67 | -.23 |
|  | Not aware | 1 | 2 | -.20 | .19 | .87 | -.65 | .25 |
|  |  | 1 | 3 | -.69* | .22 | .01 | -1.22 | -.16 |
|  |  | 2 | 3 | -.49* | .16 | .01 | -.88 | -.09 |
| Inability to approach others | Aware | 1 | 2 | -.66* | .09 | <.001 | -.88 | -.43 |
|  |  | 1 | 3 | -.97* | .11 | <.001 | -1.23 | -.72 |
|  |  | 2 | 3 | -.32* | .08 | <.001 | -.50 | -.13 |
|  | Not aware | 1 | 2 | -.05 | .17 | 1.00 | -.45 | .36 |
|  |  | 1 | 3 | -.38 | .19 | .14 | -.84 | .08 |
|  |  | 2 | 3 | -.34* | .14 | .05 | -.67 | .00 |
| Family conflict | Aware | 1 | 2 | -.22* | .05 | <.001 | -.35 | -.09 |
|  |  | 1 | 3 | -.37* | .07 | <.001 | -.54 | -.20 |
|  |  | 2 | 3 | -.15* | .06 | .03 | -.29 | -.01 |
|  | Not aware | 1 | 2 | -.08 | .10 | 1.00 | -.32 | .15 |
|  |  | 1 | 3 | -.24 | .13 | .16 | -.55 | .06 |
|  |  | 2 | 3 | -.16 | .10 | .35 | -.41 | .09 |
| Loss of institutional support for the child | Aware | 1 | 2 | -.76* | .09 | <.001 | -.98 | -.54 |
|  |  | 1 | 3 | -1.20* | .12 | <.001 | -1.50 | -.90 |
|  |  | 2 | 3 | -.45* | .11 | <.001 | -.71 | -.19 |
|  | Not aware | 1 | 2 | -.34 | .16 | .11 | -.73 | .05 |
|  |  | 1 | 3 | -.80* | .22 | .001 | -1.34 | -.26 |
|  |  | 2 | 3 | -.46 | .19 | .06 | -.92 | .01 |
| *Note.* Pairwise comparisons based on estimated marginal means of parent ratings of child concerns. Multiple comparisons were adjusted using the Bonferroni method. Time point 1: Before COVID-19; Time point 2: At the start of COVID-19; Time point 3: During COVID-19 (at the time of survey completion).  *The mean difference is significant at the .05 level. | | | | | | | | |
